# Supplementary material for: Crystal structures of Ryanodine Receptor reveal dantrolene and azumolene interactions guiding inhibitor development
Source: Nat Commun. 2025 Nov 18;16:10110. doi: 10.1038/s41467-025-65096-1 (PMC12627627; doi:10.1038/s41467-025-65096-1)
Supplement: Supplementary file 1 — Supplementary Information [file 41467_2025_65096_MOESM1_ESM.pdf]

Supplementary Information

Crystal structures of Ryanodine Receptor reveal dantrolene and azumolene interactions guiding inhibitor development

Hadiatullah Hadiatullah[1#], Lianyun Lin[1#], Zhiyan Wang[2#], Rajamanikandan Sundarraj[1], Qing Wang[1], Xinru Lai[1], Nagomi Kurebayashi[3], Takuya Kobayashi[3], Toshiko Yamazawa[4], Yu Seby Chen[5], Wenlan Wang[1], Hongxia Zhao[1], Yiqing Yin[6], Takashi Murayama[3], Filip Van Petegem[5], Zhiguang Yuchi[1,2,7,8✉]

[1]State Key Laboratory of Synthetic Biology and Frontiers Science Center for Synthetic Biology; Tianjin Key Laboratory for Modern Drug Delivery & High-Efficiency; School of Pharmaceutical Science and Technology, Faculty of Medicine, Tianjin University, Tianjin, China

[2]Department of Molecular Pharmacology, Tianjin Medical University Cancer Institute & Hospital; National Clinical Research Center for Cancer; Key Laboratory of Cancer Prevention and Therapy; Tianjin's Clinical Research Center for Cancer, Tianjin, China

[3]Department of Pharmacology, Juntendo University School of Medicine, Tokyo, Japan

[4]Core Research Facilities, The Jikei University School of Medicine, Tokyo, Japan

[5]Department of Biochemistry and Molecular Biology, Life Sciences Institute, University of British Columbia, Vancouver, British Columbia, Canada

[6]Department of Molecular Pharmacology, Department of Anesthesiology, Tianjin Medical University Cancer Institute & Hospital; National Clinical Research Center for Cancer; Key Laboratory of Cancer Prevention and Therapy; Tianjin's Clinical Research Center for Cancer, Tianjin, China

[7]Haihe Laboratory of Sustainable Chemical Transformations, Tianjin, China

[8]Guangdong Laboratory for Lingnan Modern Agriculture (Shenzhen Branch), Agricultural Genomics Institute at Shenzhen, Chinese Academy of Agricultural Sciences, Shenzhen, Guangdong, China

✉email: yuchi@tju.edu.cn

# These authors contributed equally

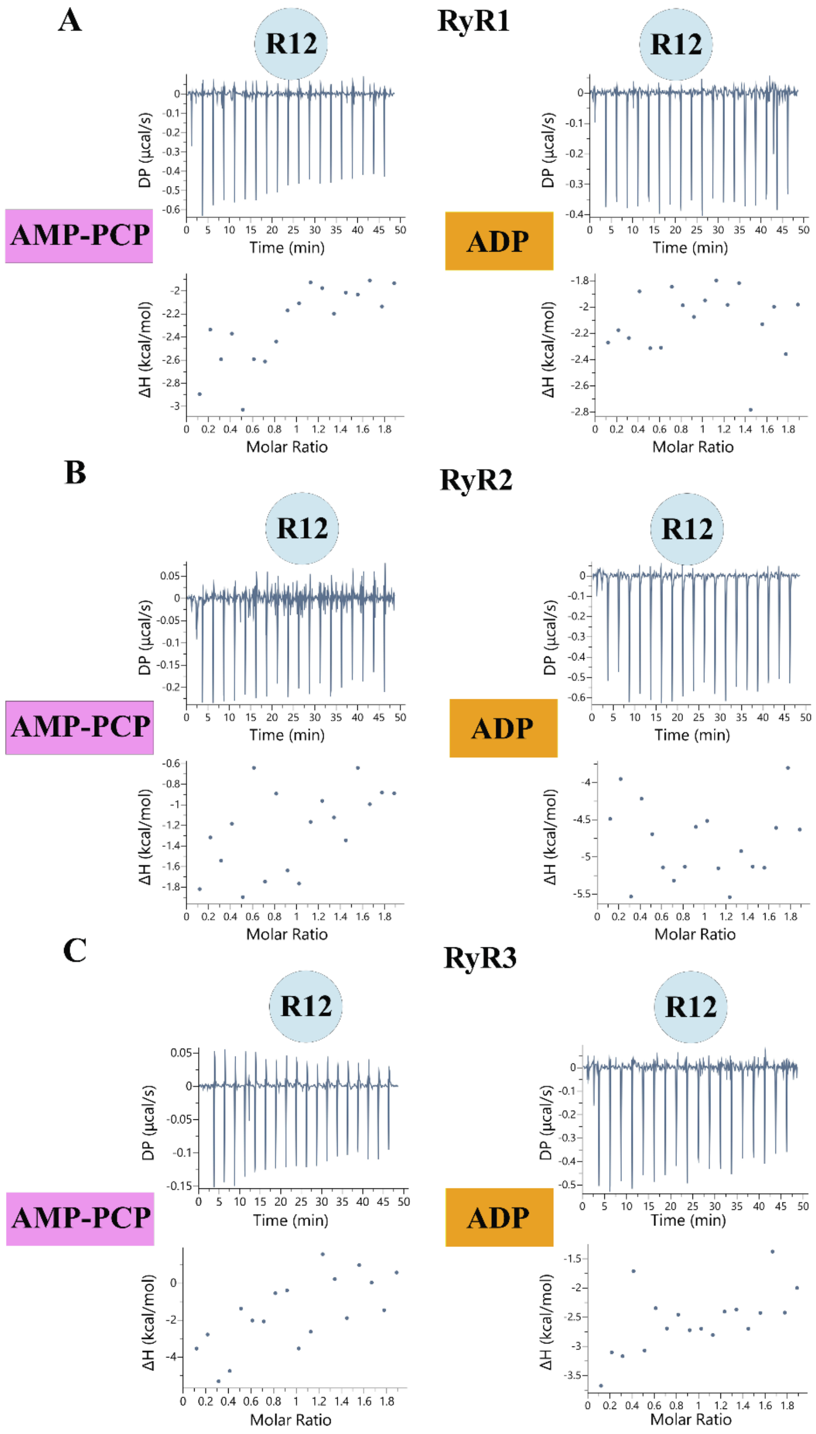

Supplementary Figure 1. ITC binding isotherms illustrating the interactions of AMP-PCP/ADP with the R12 domain of RyR1 (A), RyR2 (B), and RyR3 (C).

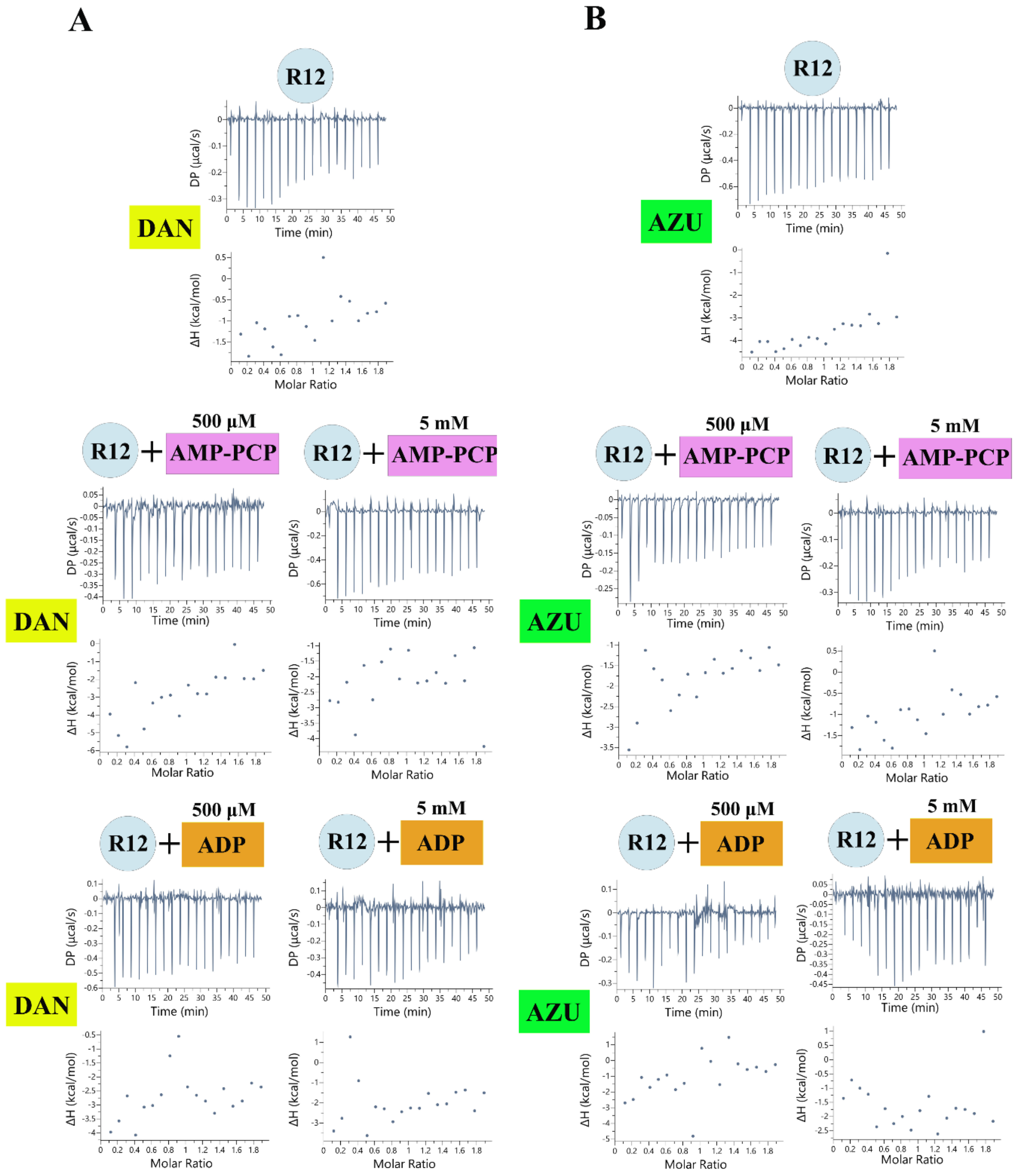

Supplementary Figure 2. ITC binding isotherms illustrating the interaction of DAN (A) or AZU (B) with the R12 domain of RyR2 in the absence or presence of AMP-PCP or ADP. The affinity and thermodynamic parameters are listed in Supplementary Table 1.

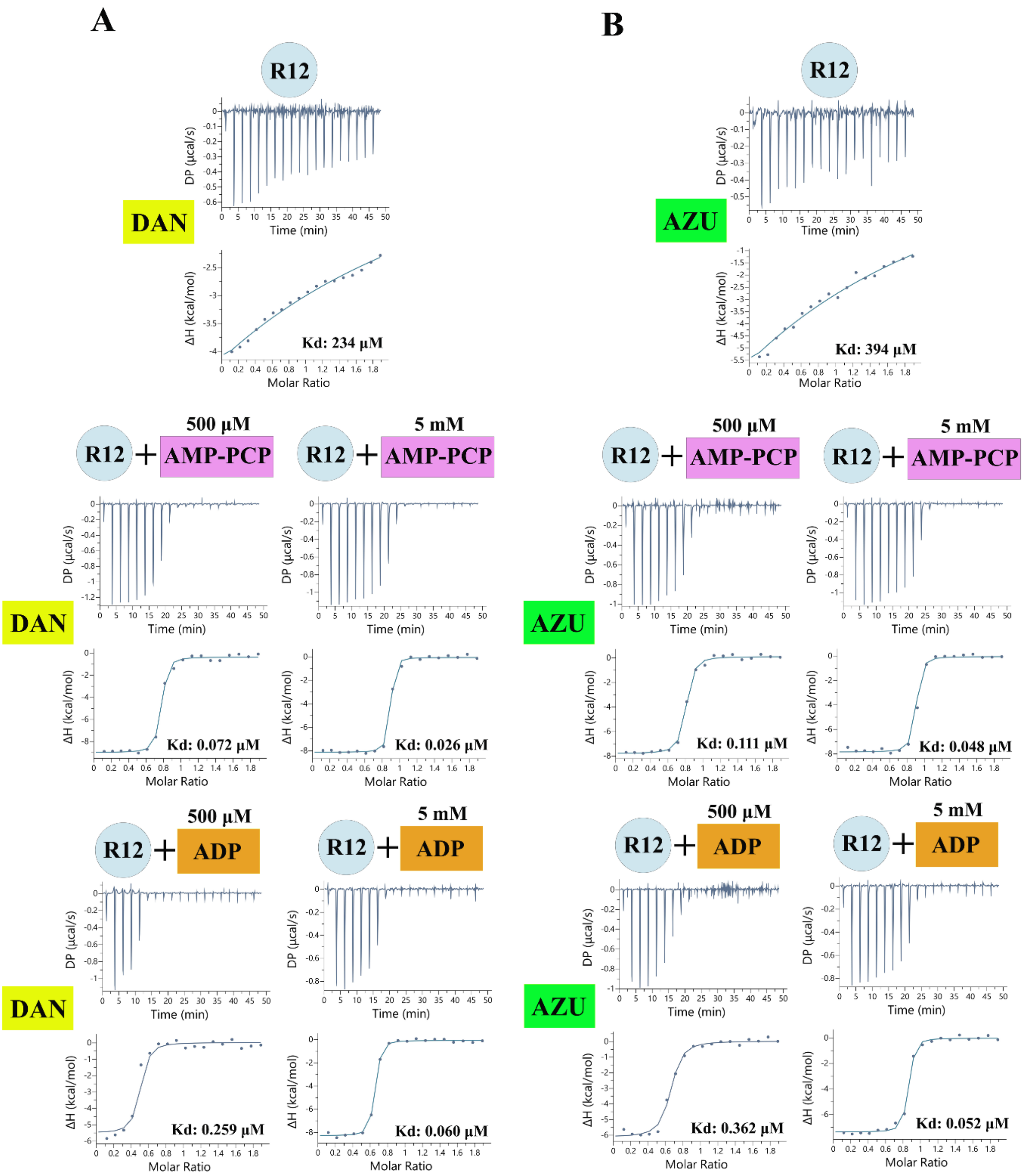

Supplementary Figure 3. ITC binding isotherms illustrating the interaction of DAN (A) or AZU (B) with the R12 domain of RyR3 in the absence or presence of AMP-PCP or ADP. The affinity and thermodynamic parameters are listed in Supplementary Table 1.

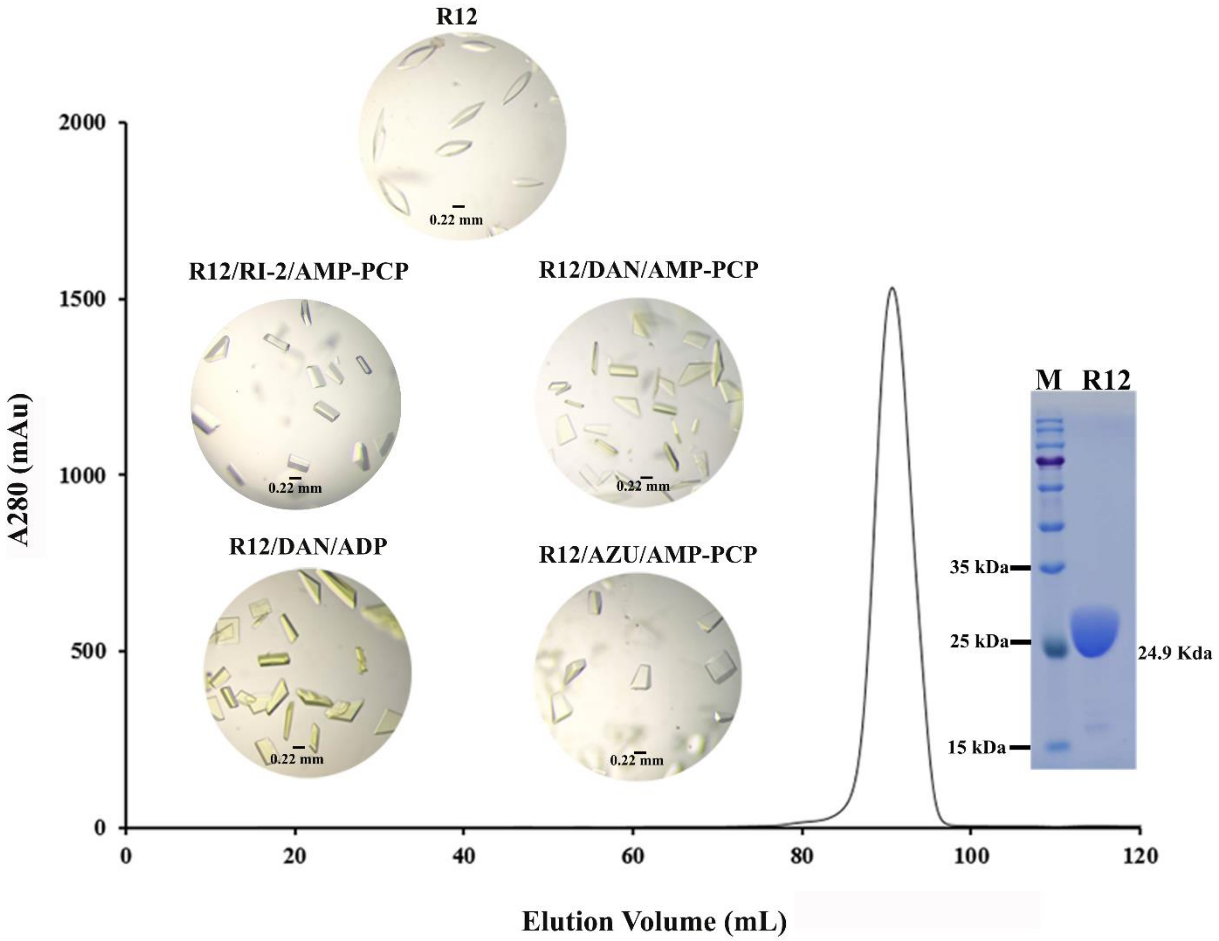

Supplementary Figure 4. Purification and crystallization of the RyR3 R12 domain. Size exclusion chromatography elution profile of the purified RyR3 R12 protein using a Superdex 200 16/600 column (GE Healthcare). The inset shows a representative 15% SDS-PAGE gel of the purified protein and representaive crystals of the apo form and ternary complexes of the RyR3 R12 domain.

A

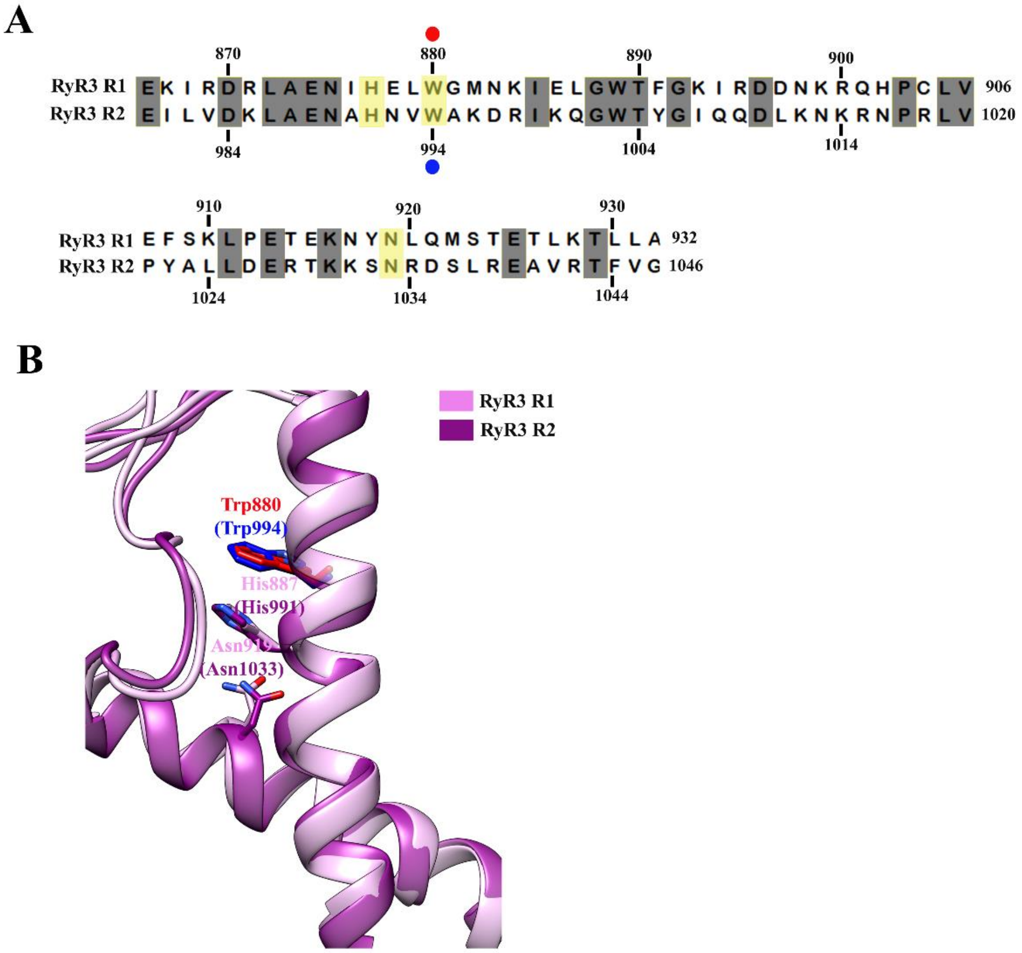

Supplementary Figure 5. Pseudo-twofold symmetry of the R12 domain. (A) Sequence alignment between the R1 and R2 regions of RyR3. Conserved residues are shaded in black, and ligand-coordinating residues are highlighted in yellow. (B) Structural superposition of the R1 region (plump) and R2 region (dark magenta) of RyR3. Side chains of conserved residues involved in ligand binding are shown, with two key tryptophan residues highlighted in red (Trp880) and blue (Trp994).

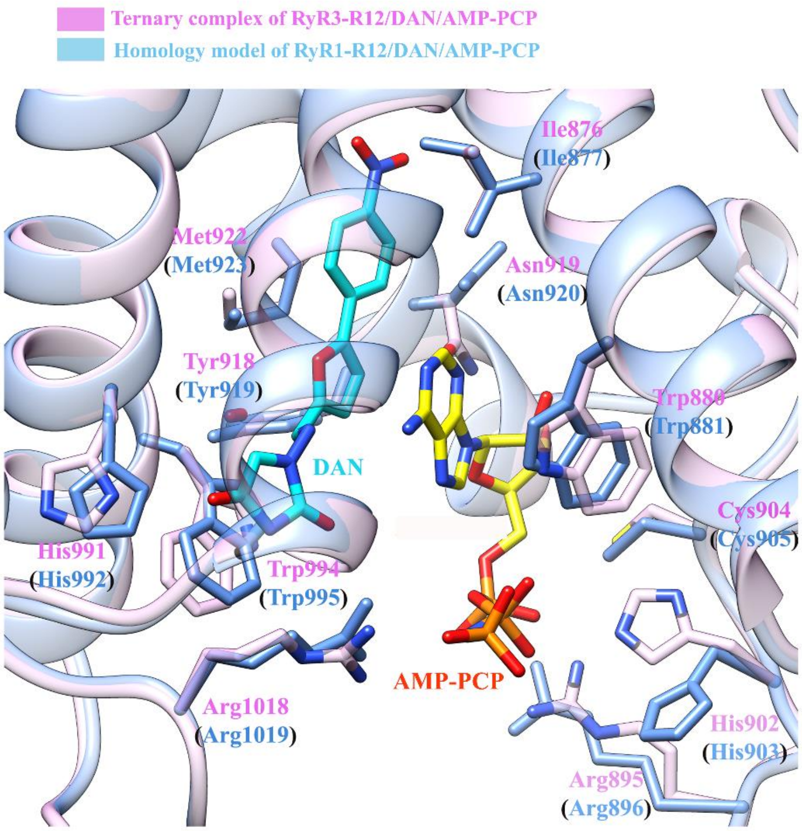

Supplementary Figure 6. Superposition of the RyR1 and RyR3 R12 domains. The crystal structure of RyR3-R12/DAN/AMP-PCP (plump) was superposed with the homology model of RyR1-R12 (light blue). Key ligand-coordinating residues are shown as sticks.

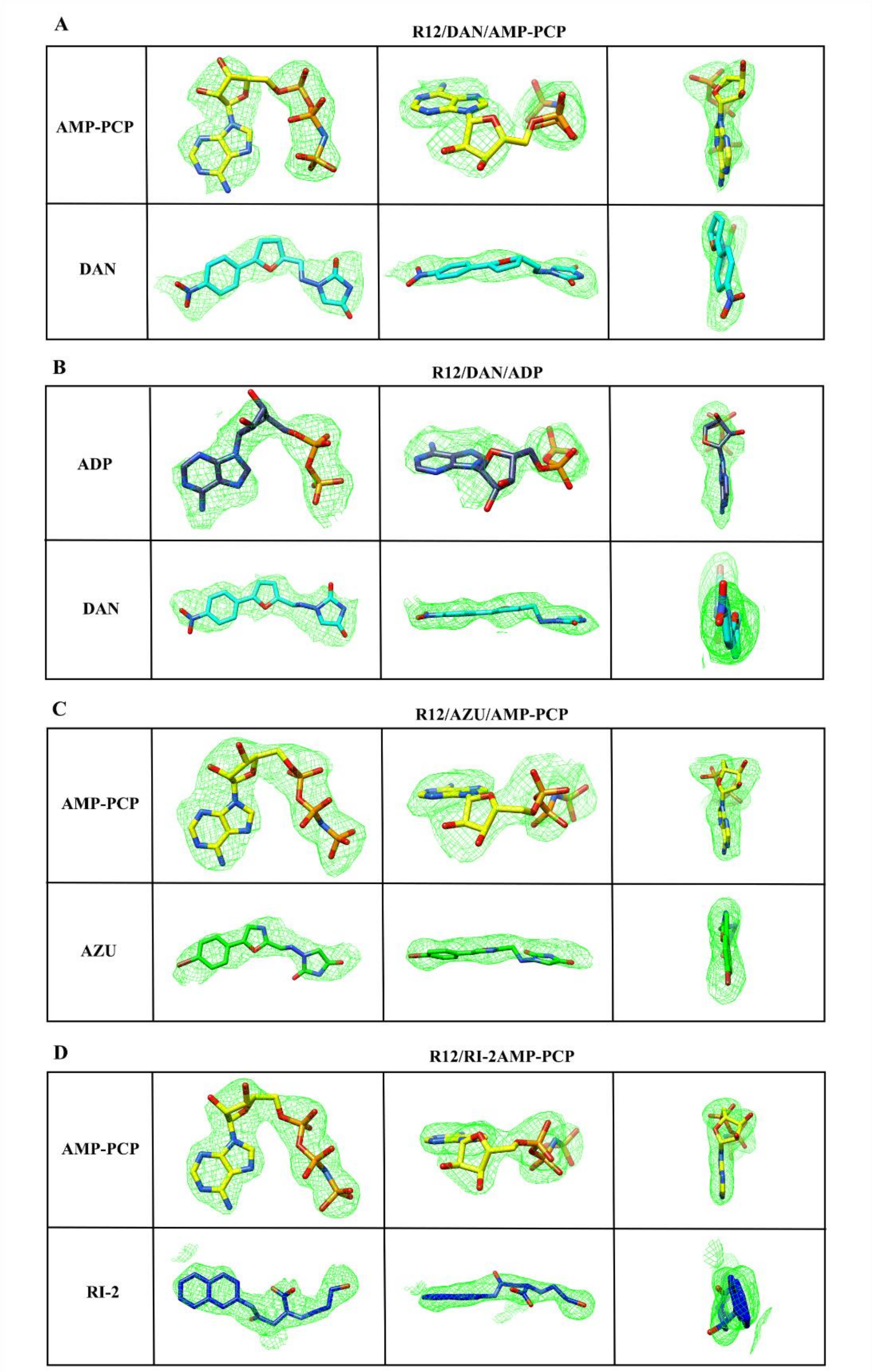

Supplementary Figure 7. Omit electron density maps for the ligands. Shown are the omit maps for ligands from the crystal structures of (A) R12/DAN/AMP-PCP, (B) R12/DAN/ADP, (C) R12/AZU/AMP-PCP, and (D) R12/RI-2/AMP-PCP, contoured at 1σ. Composite OMIT maps were generated using PHENIX (version 1.18.2-3874).

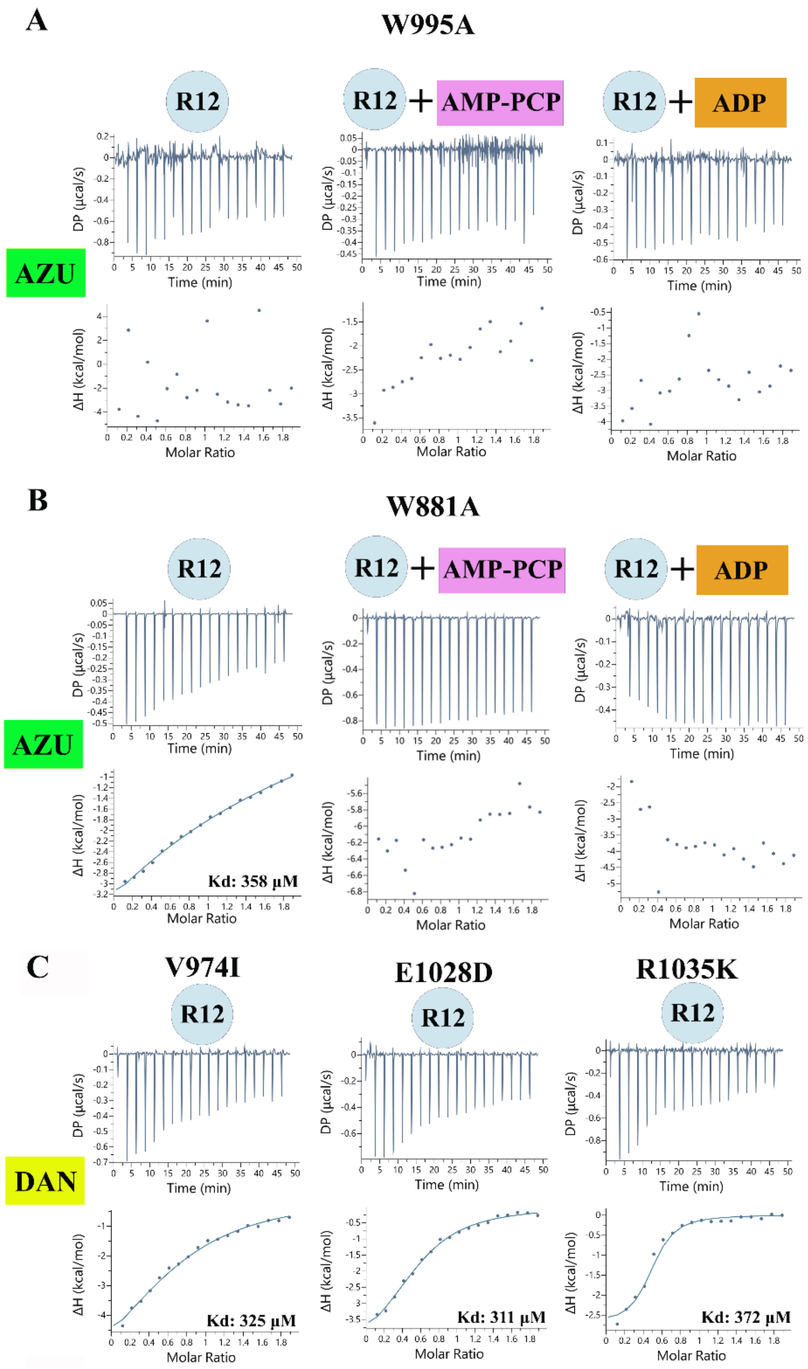

Supplementary Figure 8. Validation of Key Residues for Ligand Binding of AZU. (A) ITC binding isotherms showing the interaction of AZU with the R12 domain of RyR1 harboring the W995A and (B) W881A mutations. (C) ITC binding isotherms showing the interaction of DAN with the R12 domain of RyR1 harboring the V974I, E1028D, and R1035K. The affinity and thermodynamic parameters are listed in Supplementary Table 1.

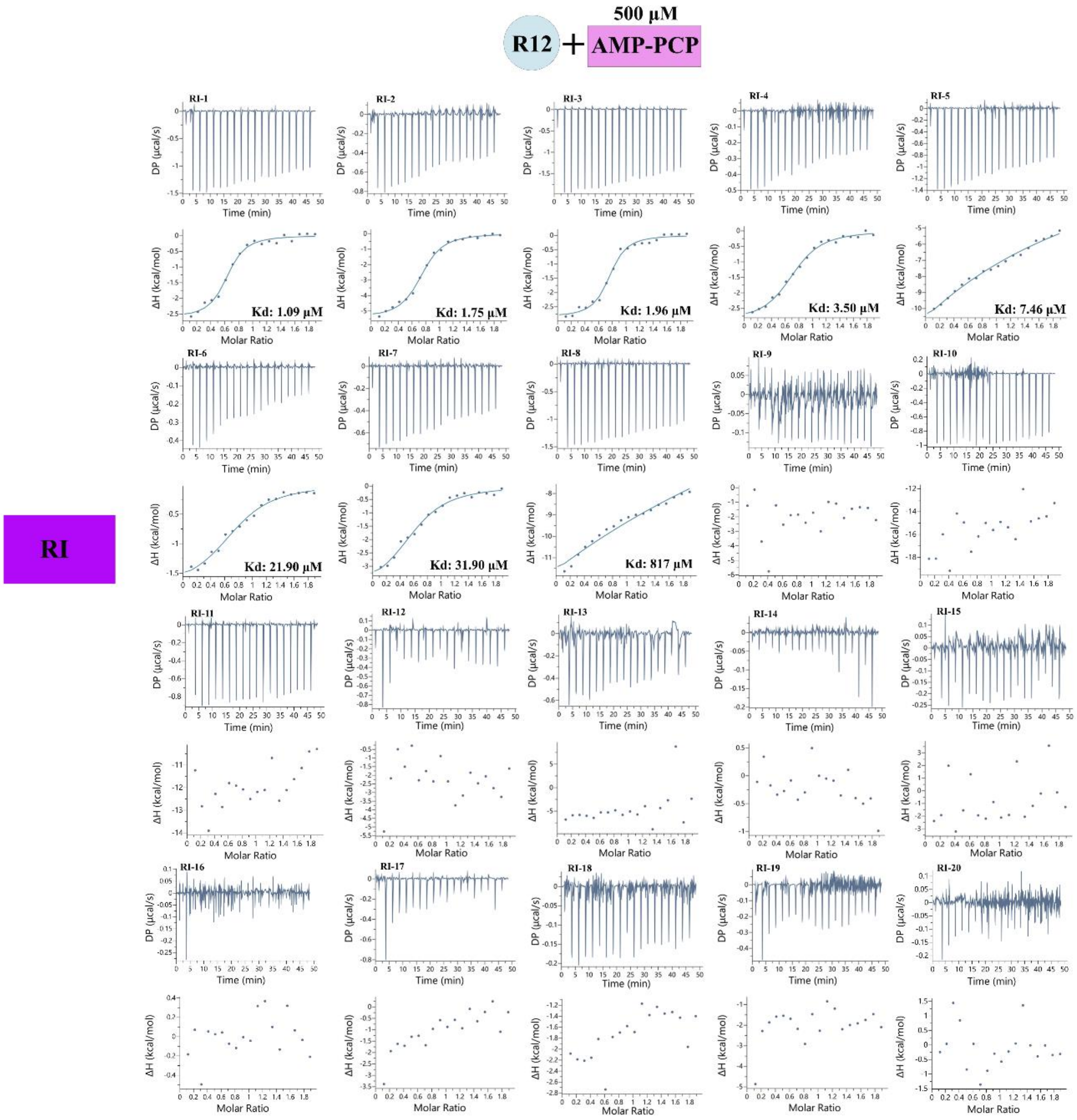

Supplementary Figure 9. Validation of Top 20 compounds using ITC.

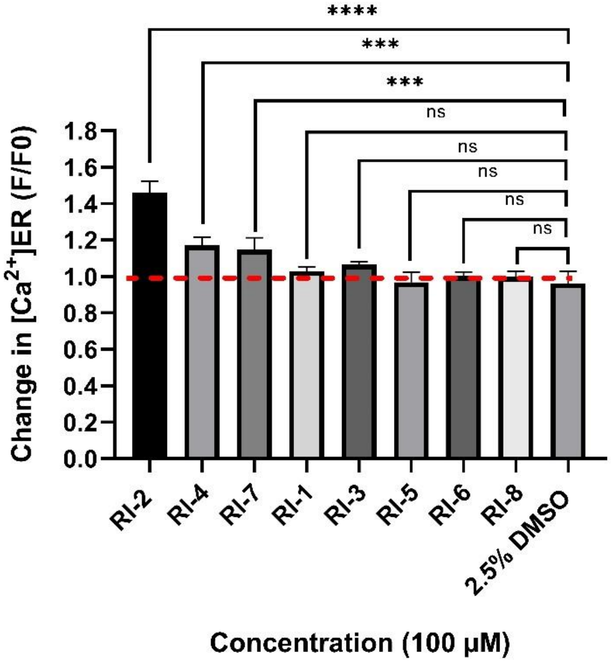

Supplementary Figure 10. Validation of the 8 selected compounds using time-lapse [Ca^{2+}] ER measurements. Data are presented as the mean ± SD (n = 3 for each group). ***$p < 0.001$, ****, $p<0.0001$ compared with 2.5% DMSO (one-way ANOVA with Dunnett's test).

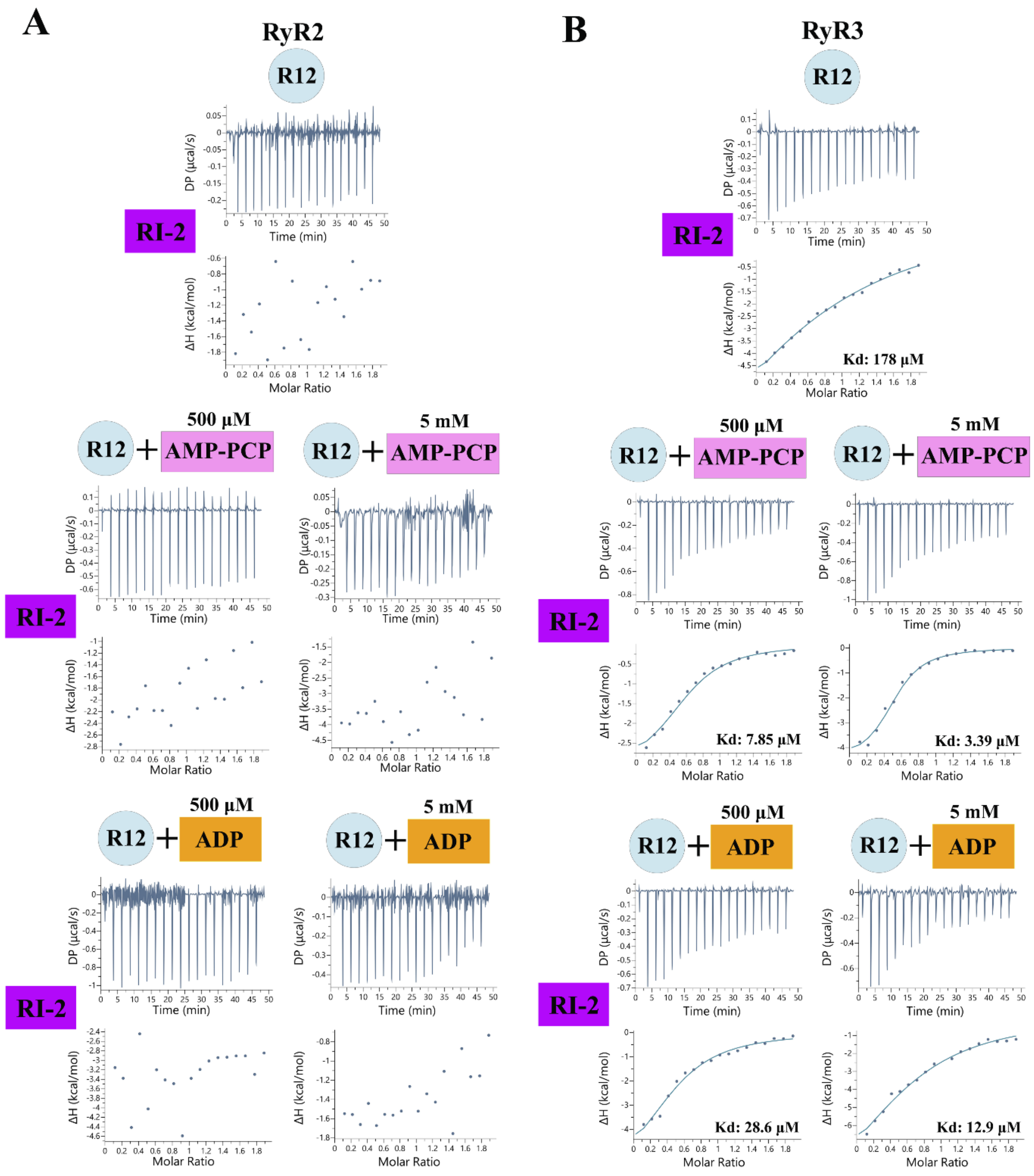

Supplementary Figure 11. ITC binding isotherms illustrating the interaction of RI-2 with the R12 domain of RyR2 (A) and RyR3 (B) in the absence or presence of AMP-PCP or ADP. The affinity and thermodynamic parameters are listed in Supplementary Table 1.

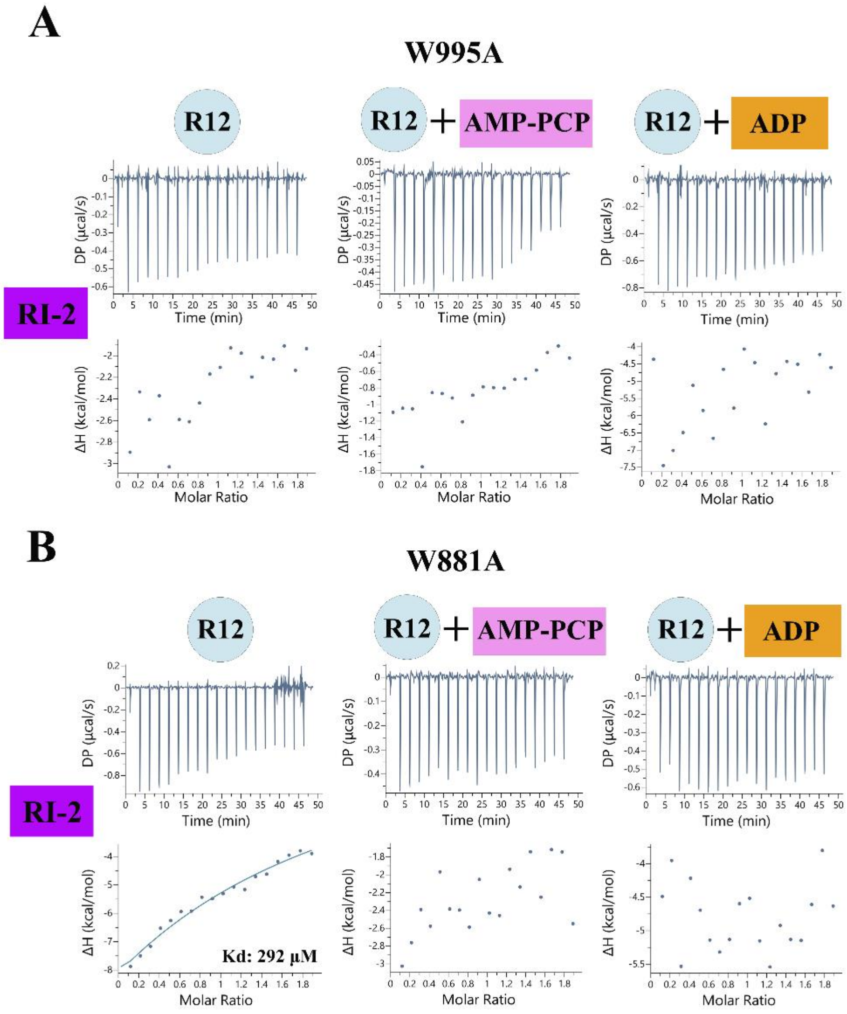

Supplementary Figure 12. Validation of Key Residues for Ligand Binding and Isoform Specificity of RI-2. ITC binding isotherms showing the interaction of RI-2 with the R12 domain of RyR1 harboring the W995A (A) and W881A (B) mutations. The affinity and thermodynamic parameters are listed in Supplementary Table 1.

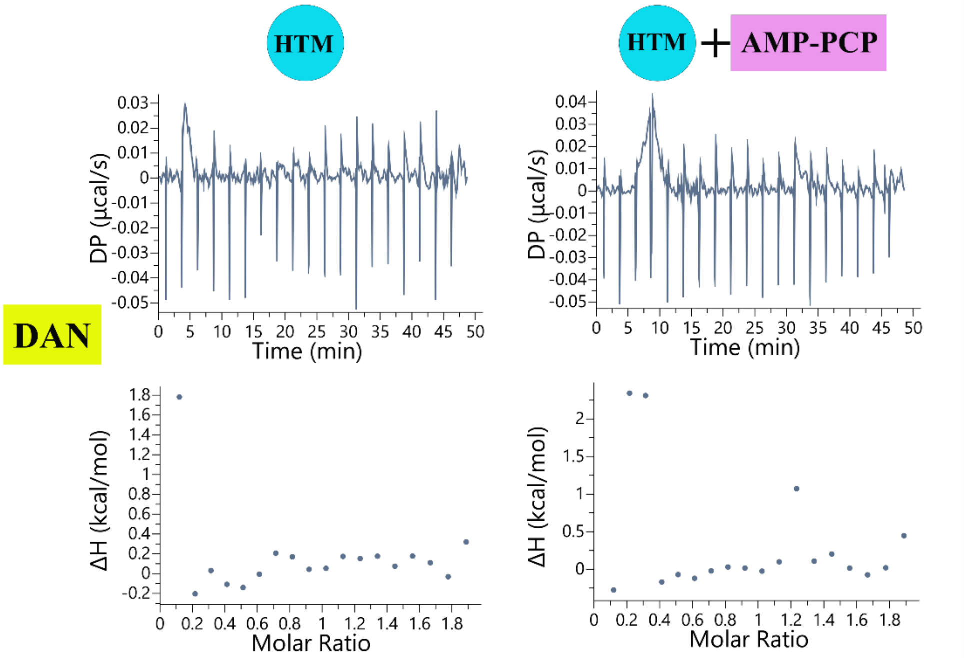

Supplementary Figure 13. ITC binding isotherms illustrating the interactions of DAN with the hexahistidine-tagged MBP (HTM) without R12 in the absent (left) and presence (right) of AMP-PCP.

Supplementary Table 1. Thermodynamic parameters of binding between DAN/AZU/RI-2 and R12 in the absence or presence of AMP-PCP or ADP.

Isoform	Titrant	Titrate	Kd (µM)	ΔH (kcal/ mol)	−TΔS (kcal/mol)	No of Sites (N)
RyR1	DAN	R12	343.1 ± 13.3	-80	74.90	0.913 ± 0.51
	DAN/500 µM AMP-PCP	R12/500 µM AMP-PCP	0.099 ± 0.014	-8.18 ± 0.79	-1.37	0.625 ± 0.28
	DAN/5 mM AMP-PCP	R12/5 mM AMP-PCP	0.022 ± 0.006	-8.24 ± 0.84	-2.20	0.825 ± 0.31
	DAN/500 µM ADP	R12/500 µM ADP	0.124 ± 0.033	-8.08 ± 0.14	-1.34	0.570 ± 0.49
	DAN/5 mM ADP	R12/5 mM ADP	0.062 ± 0.009	-8.41 ± 0.63	-1.42	0.768 ± 0.21
	AZU	R12	595.9 ± 0.012	-80	75.60	0.810 ± 0.21
	AZU/500 µM AMP-PCP	R12/500 µM AMP-PCP	0.114 ± 0.530	-2.99 ± 0.47	-6.48	0.904 ± 0.52
	AZU/5 mM AMP-PCP	R12/5 mM AMP-PCP	0.046 ± 0.014	-7.75 ± 0.12	-2.26	0.718 ± 0.50
	AZU/500 µM ADP	R12/500 µM ADP	0.241 ± 0.031	-7.92 ± 0.91	-1.11	0.652 ± 0.16
	AZU/5 mM ADP	R12/5 mM ADP	0.060 ± 0.012	-8.45 ± 0.94	-1.40	0.489 ± 0.28
RyR2	DAN	R12	-	-	-	-
	DAN/500 µM AMP-PCP	R12/500 µM AMP-PCP	-	-	-	-
	DAN/5 mM AMP-PCP	R12/5 mM AMP-PCP	-	-	-	-
	DAN/500 µM ADP	R12/500 µM ADP	-	-	-	-
	DAN/5 mM ADP	R12/5 mM ADP	-	-	-	-
	AZU	R12	-	-	-	-
	AZU/500 µM AMP-PCP	R12/500 µM AMP-PCP	-	-	-	-
	AZU/5 mM AMP-PCP	R12/5 mM AMP-PCP	-	-	-	-
	AZU/500 µM ADP	R12/500 µM ADP	-	-	-	-
	AZU/5 mM ADP	R12/5 mM ADP	-	-	-	-
RyR3	DAN	R12	234.6 ± 121.9	-80	75.60	0.651 ± 0.11
	DAN/500 µM AMP-PCP	R12/500 µM AMP-PCP	0.072 ± 0.020	-8.63 ± 0.14	-1.11	0.726 ± 0.53
	DAN/5 mM AMP-PCP	R12/5 mM AMP-PCP	0.026 ± 0.010	-8.07± 0.10	-2.27	0.842 ± 0.36
	DAN/500 µM ADP	R12/500 µM ADP	0.259 ± 0.031	-5.53 ± 0.34	-3.46	0.461 ± 0.26
	DAN/5 mM ADP	R12/5 mM ADP	0.060 ± 0.009	-8.21 ± 0.75	-1.64	0.606 ± 0.23
	AZU	R12	394 ± 7.05	-80	75.40	1.02 ± 0.41
	AZU/500 µM AMP-PCP	R12/500 µM AMP-PCP	0.111 ± 0.025	-7.90 ± 0.11	-1.59	0.760 ± 0.45
	AZU/5 mM AMP-PCP	R12/5 mM AMP-PCP	0.048 ± 0.021	-7.80 ± 0.15	-2.18	0.854 ± 0.21
	AZU/500 µM ADP	R12/500 µM ADP	0.362 ± 0.086	-6.16 ± 0.15	-2.63	0.613 ± 0.82
	AZU/5 mM ADP	R12/ADP (5 mM)	0.052 ± 0.013	-7.35 ± 0.11	-2.59	0.813 ± 0.48
RyR1-W995A	DAN	R12	-	-	-	-
	DAN/AMP-PCP (500 µM)	R12/AMP-PCP (500 µM)	-	-	-	-
	DAN/ADP (500 µM)	R12/ADP (500 µM)	-	-	-	-
	AZU	R12	-	-	-	-
	AZU/AMP-PCP (500 µM)	R12/AMP-PCP (500 µM)	-	-	-	-

	AZU/ADP (500 µM)	R12/ADP (500 µM)	-	-	-	-
RyR1-W881A	DAN	R12	313 ± 6.25	-80	75.20	0.856 ± 0.21
	DAN/AMP-PCP (500 µM)	R12/AMP-PCP (500 µM)	-	-	-	-
	DAN/ADP (500 µM)	R12/ADP (500 µM)	-	-	-	-
	AZU	R12	358 ± 138	-35.60	30.90	1.04 ± 0.35
	AZU/AMP-PCP (500 µM)	R12/AMP-PCP (500 µM)	-	-	-	-
	AZU/ADP (500 µM)	R12/ADP (500 µM)	-	-	-	-
RyR1-L969M	DAN	R12	-	-	-	-
RyR1-L983M	DAN	R12	-	-	-	-
RyR1-V974I	DAN	R12	325 ± 103	-7.56 ± 2.04	1.29	0.709 ± 0.41
RyR1-E1028D	DAN	R12	311 ± 152	-4.87 ± 0.49	1.97	0.558 ± 0.23
RyR1-R1035K	DAN	R12	372 ± 171	-2.75 ± 0.17	5.12	0.472 ± 0.11
RyR1	RI-2	R12	158.6 ± 39.8	-5.61 ± 0.73	0.944	0.519 ± 0.25
	RI-2/AMP-PCP (500 µM)	R12/AMP-PCP (500 µM)	1.75 ± 0.29	-5.47 ± 0.17	-2.39	0.721 ± 0.12
	RI-2/AMP-PCP (5 mM)	R12/AMP-PCP (5 mM)	0.56 ± 0.17	-7.05 ± 0.69	-0.11	0.547 ± 0.43
	RI-2/ADP (500 µM)	R12/ADP (500 µM)	49.10 ± 50.1	-3.08 ± 0.20	-4.30	0.605 ± 0.21
	RI-2/ADP (5 mM)	R12/ADP (5 mM)	3.86 ± 0.975	-15.09 ± 11.60	10.1	0.912 ± 0.43
RyR2	RI-2	R12	-	-	-	-
	RI-2/AMP-PCP (500 µM)	R12/AMP-PCP (500 µM)	-	-	-	-
	RI-2/AMP-PCP (5 mM)	R12/AMP-PCP (5 mM)	-	-	-	-
	RI-2/ADP (500 µM)	R12/ADP (500 µM)	-	-	-	-
	RI-2/ADP (5 mM)	R12/ADP (5 mM)	-	-	-	-
RyR3	RI-2	R12	178.2 ± 93.1	-16.2 ± 16.70	10.6	0.970 ± 0.19
	RI-2/AMP-PCP (500 µM)	R12/AMP-PCP (500 µM)	7.85 ± 2.69	-3.25 ± 0.40	-3.72	0.604 ± 0.31
	RI-2/AMP-PCP (5 mM)	R12/AMP-PCP (5 mM)	3.39 ± 0.83	-4.58 ± 0.28	-2.89	0.508 ± 0.21
	RI-2/ADP (500 µM)	R12/ADP (500 µM)	28.60 ± 18.70	-6.40 ± 1.66	-0.27	0.509 ± 0.54
	RI-2/ADP (5 mM)	R12/ADP (5 mM)	12.90 ± 6.20	-11.9 ± 4.87	5.75	0.694 ± 0.42
RyR1-W995A	RI-2	R12	-	-	-	-
	RI-2/AMP-PCP (500 µM)	R12/AMP-PCP (500 µM)	-	-	-	-
	RI-2/ADP (500 µM)	R12/ADP (500 µM)	-	-	-	-
RyR1-W881A	RI-2	R12	292 ± 22.5	-10.7 ± 3.83	4.71	0.94 ± 0.46
	RI-2/AMP-PCP (500 µM)	R12/AMP-PCP (500 µM)	-	-	-	-
	RI-2/ADP (500 µM)	R12/ADP (500 µM)	-	-	-	-

Supplementary Table 2. Data collection and refinement statistics for the R12 Crystals.

Data collection	R12	R12/DAN/AMP-PCP	R12/DAN/ ADP	R12/AZU/AMP-PCP	R12/RI-2/AMP-PCP
λ for data collection (Å)	0.9795	0.9795	0.9795	0.9795	0.9795
PDB ID	9L92	9L90	9L9B	9L91	9LS7
**Data collection**					
Space group	P 1 21 1	P 21 21 21	P 21 21 21	P 21 21 21	P 21 21 21
Cell dimension (Å)					
a, b, c (Å)	40.04| 122.15| 47.71	60.74| 60.84| 262.52	62.04| 61.85| 279.11	59.96 | 60.76| 261.67	60.35| 61.61| 277.58
α, β, γ, (°)	90.00, 93.27, 90.00	90.00, 90.00, 90.00	90.00, 90.00, 90.00	90.00, 90.00, 90.00	90.00, 90.00, 90.00
Resolution (Å)	47.63-1.97	131.26-2.79	139.60-2.84	130.83-3.00	138.79-2.49
R-merge	0.07 (0.74)	0.07 (0.82)	0.19 (0.78)	0.10 (0.84)	0.16 (0.96)
CC1/2	0.99 (0.76)	0.99 (0.79)	0.99 (0.65)	0.99 (0.90)	0.99 (0.95)
Completeness (%)	99.70 (98.90)	99.80 (99.96)	95.65 (69.27)	96.32 (96.78)	92.07 (78.29)
Redundancy	6.6 (5.7)	6.5 (6.0)	9.8 (9.2)	6.7 (6.0)	8.3 (8.0)
Average I/σ(I)	19.7 (2.6)	13.2 (1.8)	8.9 (2.3)	13.4 (2.4)	8.2 (1.4)
**Refinement**					
Resolution (Å)	47.63-1.97 (2.03-1.97)	131.26-2.79	139.60-2.84	65.42.80-3.00	16.96-2.49
		(2.89-2.79)	(2.94-2.84)	(3.10-3.00)	(2.58-2.49)
No. of reflections	32463 (3208)	25119 (2487)	25082 (1535)	19967 (1926)	29267 (1483)
Rwork	0.229	0.272	0.280	0.261	0.232
Rfree	0.278	0.319	0.315	0.310	0.270
RMSD bonds (Å)	0.015	0.006	0.009	0.005	0.002
RMSD angle (°)	1.99	1.51	1.63	1.06	0.47
No. of atoms					

Protein	404	810	814	819	809
Ligands	24	108	100	104	108
Water	0	23	15	49	66
Ramachandran plot (%)					
Most favored	95.48	94.35	93.50	96.41	96.99
Additionally allowed	4.02	5.65	6.50	3.47	3.01
Outliers	0.50	0.00	0.00	0.12	0.00

Supplementary Table 3. Primers for RyR1 R12 mutations used for ITC.

Name	Sequence (5'→ 3')
RyR1-R12-W881A-F	AATATTCATGAACTGGCGGCGCTGACCCGCATT
RyR1-R12-W881A-R	AATGCGGGTCAGCGCCGCCAGTTCATGAATATT
RyR1-R12-W995A-F	AACGGCCATAACGTGGCGGCGCGCGATCGCGTG
RyR1-R12-W995A-R	CACGCGATCGCGCGCCGCCACGTTATGGCCGTT
RyR1-R12-L969M-F	TATAAACCGGCGCCGATGGATCTGAGCCATGT
RyR1-R12-L969M-R	ACATGGCTCAGATCCATCGGCGCCGGTTTATA
RyR1-R12-V974I-F	CTGGATCTGAGCCATATACGCCTGACCCCG
RyR1-R12-V974I-R	CGGGGTCAGGCGTATATGGCTCAGATCCAG
RyR1-R12-L983M-F	GCGCAGACCACCATGGTGGATCGCCTGGCG
RyR1-R12-L983M-R	CGCCAGGCGATCCACCATGGTGGTCTGCGC
RyR1-R12-E1028D-F	TATCGCCTGCTGGATGATGCGACCAAACGC
RyR1-R12-E1028D-R	GCGTTTGGTCGCATCATCCAGCAGGCGATA
RyR1-R12-R1035K-F	ACCAAACGCAGCAACAAGGATAGCCTGTGCCAA
RyR1-R12-R1035K-R	TTGGCACAGGCTATCCTTGTTGCTGCGTTTGGT
