# Supplementary material for: Crystal structures of Ryanodine Receptor reveal dantrolene and azumolene interactions guiding inhibitor development
Source: Nat Commun. 2025 Nov 18;16:10110. doi: 10.1038/s41467-025-65096-1 (PMC12627627; doi:10.1038/s41467-025-65096-1)
Supplement: Supplementary file 2 — Description of Additional Supplementary Files [file 41467_2025_65096_MOESM2_ESM.docx]

Description of Additional Supplementary Files

Supplementary Data 1: Homology model of RyR1 R12

Supplementary Data 2: Homology model of RyR2 R12
